# Retraction Note: Gene expression profiling analysis of hepatocellular carcinoma

**DOI:** 10.1186/s40001-015-0120-x

**Published:** 2015-03-26

**Authors:** Deyong Kong, Heming Chen, Weiqun Chen, Shuiyi Liu, Hui Wang, Tangwei Wu, Hongda Lu, Qingzhi Kong, Xiaodong Huang, Zhongxin Lu

**Affiliations:** Department of Medical Laboratory, Central Hospital of Wuhan, Wuhan, 430014 China; Department of Cardiovascular and Thoracic Surgery, Second Xiangya Hospital, Central South University, Changsha, 410078 China; Department of Central Laboratory, Central Hospital of Wuhan, Wuhan, 430014 China; Cancer Research Institute of Wuhan, Wuhan, 430014 China; Department of Oncology, Central Hospital of Wuhan, Wuhan, 430014 China

## Retraction

The Publisher and Editor regretfully retract this article [1] because the peer-review process was inappropriately influenced and compromised. As a result, the scientific integrity of the article cannot be guaranteed. A systematic and detailed investigation suggests that a third party was involved in supplying fabricated details of potential peer reviewers for a large number of manuscripts submitted to different journals. In accordance with recommendations from COPE we have retracted all affected published articles, including this one. It was not possible to determine beyond doubt that the authors of this particular article were aware of any third party attempts to manipulate peer review of their manuscript.
